# Conformational changes in Apolipoprotein N-acyltransferase (Lnt)

**DOI:** 10.1038/s41598-020-57419-7

**Published:** 2020-01-20

**Authors:** Benjamin Wiseman, Martin Högbom

**Affiliations:** Department of Biochemistry and Biophysics, Stockholm University, Svante Arrhenius väg 16C, 10691 Stockholm, Sweden

**Keywords:** Enzymes, Structural biology, X-ray crystallography

## Abstract

Lipoproteins are important components of the cell envelope and are responsible for many essential cellular functions. They are produced by the post-translational covalent attachment of lipids that occurs via a sequential 3-step process controlled by three integral membrane enzymes. The last step of this process, unique to Gram-negative bacteria, is the N-acylation of the terminal cysteine by Apolipoprotein N-acyltransferase (Lnt) to form the final mature lipoprotein. Here we report 2 crystal forms of Lnt from *Escherichia coli*. In one form we observe a highly dynamic arm that is able to restrict access to the active site as well as a covalent modification to the active site cysteine consistent with the thioester acyl-intermediate. In the second form, the enzyme crystallized in an open conformation exposing the active site to the environment. In total we observe 3 unique Lnt molecules that when taken together suggest the movement of essential loops and residues are triggered by substrate binding that could control the interaction between Lnt and the incoming substrate apolipoprotein. The results provide a dynamic context for residues shown to be central for Lnt function and provide further insights into its mechanism.

## Introduction

The post-translational modification of proteins by the covalent attachment of lipids is found across all domains of life. In bacteria, lipoproteins are important components of the cell envelope and are responsible for many essential cellular functions including nutrient uptake, secretion, cell wall integrity, and antibiotic production^1^. In pathogenic bacteria lipoproteins are important virulence factors^2^. Pre-prolipoproteins are synthesized in the cytoplasm and translocated to the inner membrane via the Tat or Sec secretion pathways^3^. They contain an N-terminal signal peptide that contains a cysteine where lipid attachment occurs via a sequential 3-step process controlled by three essential membrane bound enzymes: Prolipoprotein diacylglyceryl transferase (Lgt), Lipoprotein signal peptidase (LspA), and Apolipoprotein N-acyltransferase (Lnt)^1^. The first step, carried out by Lgt, involves the transfer of a diacylglyceryl group from phosphatidylglycerol to what will become the N-terminal cysteine. This is followed by the cleavage of the signal peptide by LspA to form apolipoproteins^1^. The last step, unique to Gram-negative bacteria and high GC Gram-positive bacteria, is the N-acylation of the terminal cysteine by Lnt to form the final mature lipoprotein^4^. The mature lipoprotein then remains at the inner membrane or is translocated to the outer membrane by the Lol pathway^1^.

**Figure 1:**
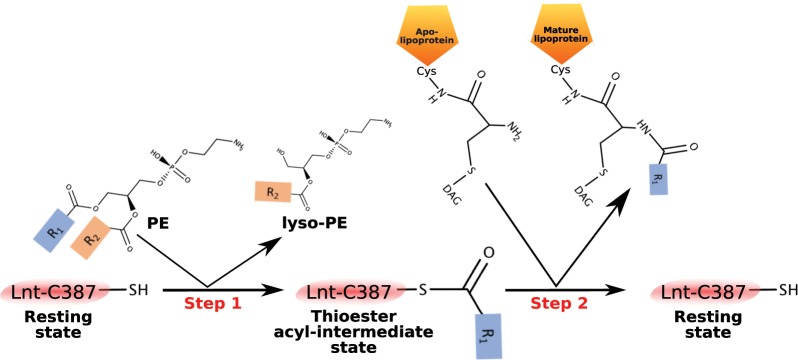
The ping-pong reaction mechanism of Lnt, where PE is its preferential lipid substrate phosphatidylethanolamine, R_1_ and R_2_ are the lipid acyl chains, and DAG is diacylglycerol. In step 1 the acyl chain is transferred from the incoming phospholipid substrate to Lnt to create a thioester linkage on the active site cysteine. In step 2 the acyl chain is transferred from this cysteine to the N-terminal cysteine of the incoming apolipoprotein.

Based on sequence similarity Apolipoprotein N-acyltransferase (Lnt) belongs to the nitrilase superfamily. Nitrilases are multimeric proteins that contain a common Glu-Lys-Cys catalytic triad that hydrolyse carbon-nitrogen bonds^5–7^. In the case of Lnt, the nitrilase domain catalyzes the attachment of a fatty acid derived from a phospholipid to the alpha-amino group of the N-terminal cysteine of the apolipoprotein creating the final mature lipoprotein. This attachment occurs via a proposed 2-step ping-pong mechanism^8,9^ (Fig. 1) where the first step is the acyl transfer of the phospholipid substrate to create a thioester linkage on the active site cysteine. The second step is the transfer of the acyl chain from this cysteine to the N-terminal cysteine of the apolipoprotein (Fig. 1). Similar to other members of the nitrilase superfamily^10^, this occurs at the catalytic triad of E267-K335-C387 where E267 acts as a general base to activate the nucleophile of the thiol group of C387 that can then attack the ester linkage between the acyl chain and the glycerol backbone of the phospholipid substrate to form the thioester acyl intermediate. K335 provides part of the oxyanion hole to stabilize this tetrahedral intermediate of the reaction^11^. It has been shown that, although the *E*. *coli* Lnt can use all available phospholipids such as phosphatidylglycerol (PG), phosphatidylethanolamine (PE), and cardiolipin (CL) as acyl donors; its preferred substrate is PE^4,12^. In the second step, with Lnt now in its thioester-acyl intermediate state, the alpha-amino group at the N-terminus of the incoming apolipoprotein attacks the thioester linkage to transfer the acyl chain to produce the final mature lipoprotein. Similar to the first step, K335 stabilizes the tetrahedral intermediate of the reaction^9^.

All three enzymes involved in lipid attachment are essential for survival in most bacteria making them attractive targets for new antimicrobial agents^13^. Recently both the structures of Lgt^14^ and LspA^15^ have been solved, and very recently a number of structures of Lnt became available^9,16,17^. Aside from the transmembrane domain, the structures of Lnt confirm that the overall fold of soluble nitrilases is conserved in Lnt. One key distinguishing feature of the Lnt nitrilase domain, however, is a long arm (residues 345–365 in *E*. *coli*) that is longer and more flexible than is seen in typical soluble nitrilases that seems to extend parallel to the membrane in most cases^18,19^. Overall, the reported structures of Lnt are all very similar and describe an enzyme essentially with or without its lipid substrate bound, with little or no information of how an incoming apolipoprotein peptide may interact with Lnt.

**Figure 2:**
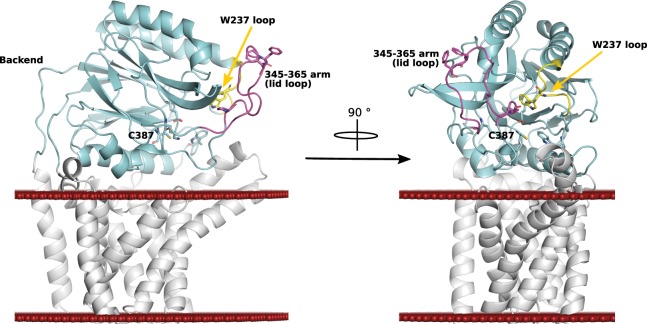
Overall structure of Lnt labelled with the key features discussed in the text. In white is the transmembrane domain; cyan the nitrilase domain; yellow the highly mobile loop containing W237; purple the highly mobile 345–365 arm. Left: rotated 90° looking directly into the large, open cavity leading to the active site C387. The red spheres represent the position of the membrane as predicted by the PPM server^34^.

The recent, near simultaneous publication of 3 papers describing the structure of Lnt were released during our crystal optimization process. Here we report the crystal structures from two constructs of the *E*. *coli* Lnt in two distinct crystallization conditions and crystal forms. Although both new forms have an overall architecture very similar to each other and to the recently reported structures with a transmembrane domain consisting of 8 helices and a soluble domain with a nitrilase fold (Fig. 2), they also contain new details that have not been reported previously. In these structures we observe two new principle findings that can be related to the second step of the Lnt reaction: (1) extra electron density extending off the active site cysteine consistent with the thioester acyl-intermediate. (2) the long 345–365 arm in a previously unseen conformation at an angle far above the membrane interface that further demonstrates its flexibility. We also observe a unique protomer arrangement in the asymmetric unit that, while likely due to crystal lattice contacts, might provide insights into the docking of an apolipoprotein onto Lnt. These results, taken together and when analyzed in the context of the other available structures^9,16,17^ and mutational studies^11,16,18,20^, reveal possible substrate induced dynamics that add important implications in active site access and the catalytic mechanism of Lnt.

## Results

As mentioned we purified and crystallized two distinct constructs of the *E*. *coli* Lnt. Construct 1 (Lnt-C1) contains a fifteen-amino acid C-terminal tail that contains the His_8_-tag used for purification. It crystallized in space group P2_1_2_1_2_1_ using the vapor-diffusion method and contains two molecules in the asymmetric unit. Construct 2 (Lnt-C2) contains only two additional residues on its N-terminus left over after cleavage of the His_6_-tag used for purification. It crystallized in space group P6_4_22 using the lipidic cubic phase (LCP) method and contains 1 molecule in the asymmetric unit.

### Restructuring of the active site is not required to accommodate the thioester acyl-intermediate

**Figure 3:**
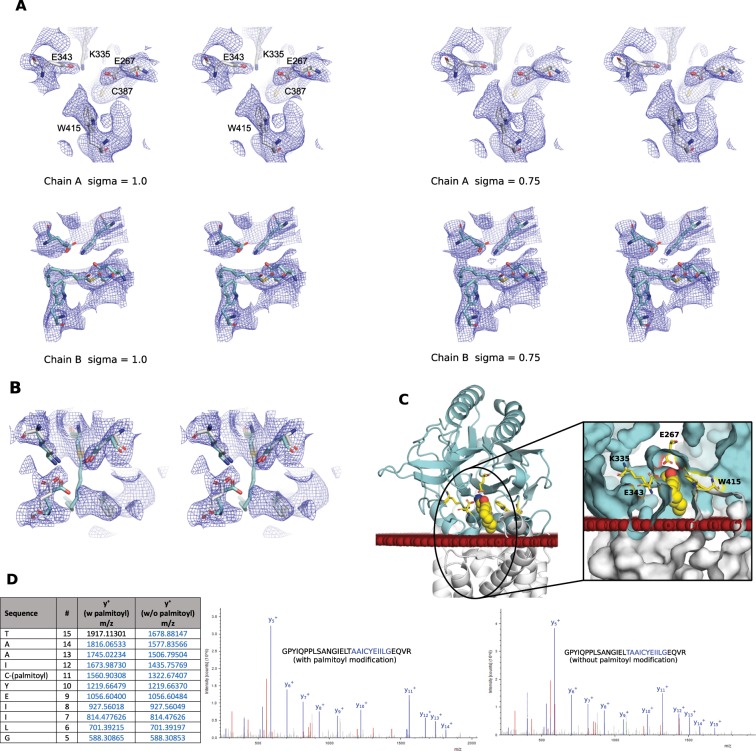
The modelled thioester acyl-intermediate of Lnt-C1. (**A**) Stereoview of the active site of chains A (white) and B (cyan) with the corresponding electron density contoured at 1.0 and 0.75 sigma levels showing the absence and presence of the extra electron density respectively. (**B**) Stereoview of an overlay of the active site of chain B onto the active site of chain A, with electron density contoured at 1.0 sigma. Cartoon (**C**) and surface (boxed) representation of the large, open substrate entry portal leading to the active site in relation to the membrane interface showing the position of the thioester intermediate (shown as spheres). The red spheres represent the position of the membrane as predicted by the PPM server^34^. (**D**) Representative MS/MS spectra for the GPYIQPPLSANGIELTAAICYEIILGEQVR peptide. Left, Containing the palmitoyl modification on C387. Right, Containing unmodified C387.

As mentioned above Lnt-C1 crystallized with 2 molecules in the asymmetric unit. In one of those molecules (chain B) clear electron density can be seen extending off the active site C387 side chain that is not seen in the other molecule, even at lower map contour levels (Fig. 3A). We interpret this extra density as the thioester acyl-intermediate based on mass spectrometry analysis (Fig. 3D) on purified Lnt that identified a palmitoyl modification at position C387. In chain A there is no density seen extending off C387, however there is unmodeled density seen extending from W415 into the active site cavity (Fig. 3B). An overlay of chain B onto chain A (Fig. 3B) places the alkyl tail of the modification near this density that could suggest the presence of a low occupancy thioester modification in chain A as well. This is also consistent with some of the other structures of Lnt (5VRG, for example) where lipid molecules are seen bound in this area, with mass spectrometry analysis indicating that a large percentage of the protein is covalently bound to palmitate^16^, and previous findings that Lnt exists as a thioester acyl-enzyme intermediate^11^. As shown in Fig. 3C, the acyl tail of the adduct is curved slightly upward and positioned above the predicted membrane interface. This can be explained by the high hydrophobicity of the binding groove^16^ that could easily accommodate hydrophobic molecules such as lipids. Furthermore, the slightly curved shape of the density is similar in shape to a palmitoylated cysteine observed in a TEAD transcription factor^21^ as well as simulations performed by Wiktor *et al*.^9^.

Despite this modification, no large movements of side chains are required to accommodate this extra density compared with the other available non-palmitoylated models. In fact, as judged by the improved density around K335 (Fig. 3A), this palmitoylation could help stabilize the active site. The catalytic cysteine, C387, of Lnt sits just above the membrane and at the back of a large open cavity (Figs. 2, 3C) that provides ample room for a palmitate molecule without the need for rearranging of the active site residues. Although mass spectrometry identified a C20-palmitoyl; we could only confidently build half of the lipid tail into electron density where the membrane region is expected to be. However, it is reasonable to assume that it extends into the membrane until the bottom of the binding pocket. Although difficult to judge at 3.5 Å resolution, there appears to be a slight movement towards the palmitate molecule of the side chain of E343 that could help further stabilize this intermediate. This residue is also located at the hinge of the flexible arm in the active site vicinity.

Mass spectrometry analysis (Fig. 3D) of both Lnt-C1 and Lnt-C2 identified unmodified as well as the palmitoyl modification at position C387. This is consistent with previous mass spectrometry analysis indicating that in a large percentage of the protein at this residue is covalently bound to palmitate^16^. The potential presence of the low occupancy thioester modification observed in chain A could be explained by its position at the back of a large open cavity and its labile nature that can lead to the slow deacylatation by hydrolytic cleavage^9^ during purification and crystallization in the absence of the second substrate. Differences between the 2 molecules within the ASU of Lnt-C1 that could account for the low amount or lack of the thioester modification in chain A compared to chain B are discussed below. This slow deacylatation also likely explains the complete lack of any extra density seen around C387 of Lnt-C2 that crystallized in an open conformation exposing the active site to the surrounding environment as well as took 6 weeks to crystallize compared to Lnt-C1 that took 2 weeks to crystallize.

### The 345–365 arm is disordered with transient secondary structured elements

**Figure 4:**
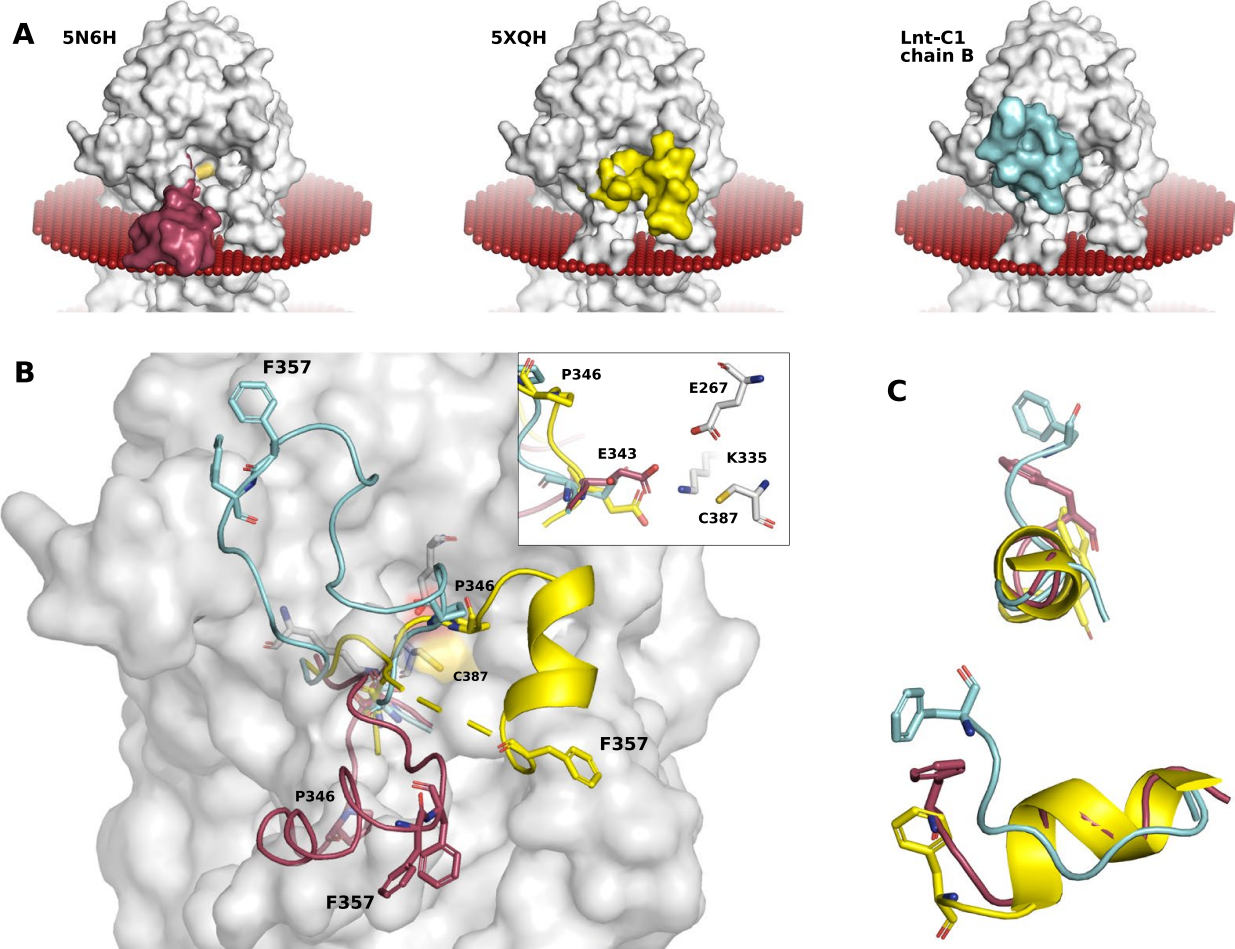
Dynamics and disordered nature of the flexible 345–365 arm. (**A**) Surface representation of Lnt-C1 chain A (grey) overlaid with the flexible loop seen in three distinct positions: positioned parallel and dipping into the membrane as seen in pdb 5N6H (maroon) opening the substrate entry portal to the environment; positioned above the membrane and across the substrate entry cavity as seen in pdb 5XHQ (yellow); and above the membrane at a ~60° angle as seen in Lnt-C1 chain B (cyan). The yellow patch is the location of the active site C387. The red spheres represent the position of the membrane as predicted by the PPM server^34^. (**B**) Surface representation of chain A overlaid with the three arm positions, same color scheme as in (**A**). For orientation the location of F357 and P346 is noted in all three arm positions. Inset: zoomed region around the active site showing the relative small movement of E343 despite the large movement of arm. (**C**) Alignment of the short helix in the flexible arm showing its unravelling. For orientation the location of F357 is shown, same color scheme as in (**A**).

One of the striking features of chain B of the Lnt-C1 structure is the position of the long 345–365 arm that is at an upward 60° angle to the membrane (Fig. 4A,B). In chain A by contrast, although residues 352–362 were not visible in the electron density, the beginning and end of the arm can be modelled extending parallel to the membrane similar to the other reported structures (pdb ids 5N6H, 5N6L, 5N6M, 5VRG, and 5VRH). When in this conformation the arm appears to be disordered and the active site is much more accessible to the surrounding environment. One consequence of this movement is a large change of position of P346 to approximately 21 Å above the membrane interface from its position when this arm is extending parallel to the interface (Fig. 4B). A previous mutational study^20^ has shown P346 to be essential for Lnt activity at higher growth temperatures. Another consequence of the mobile nature of this arm is the slight movement of the essential residue E343 that is at one of the joints allowing movement between the 2 conformations (Fig. 4B, inset), forming a salt bridge with the active site K335 when in the downward conformation, and pulling away from and breaking the salt bridge when in the upward position potentially helping to stabilize the thioester intermediate.

The positioning of this arm in chain B is made possible by an adjacent chain A protomer that makes crystal contacts with F357 and F358 that pin the arm in this upward position. Despite F357 and F358’s location at the tip of this long arm, far removed from the active site, mutational studies have previously shown F358 to be essential for Lnt activity^17,18^. Similarly, in the only other structure for which a complete arm was able to be build^9^ F357 and F358 also form crystal contacts far removed from the active site. Also, in one of the structures (pdb id 5XHQ) this arm contains a small 7 amino acid alpha-helix (residues 349–355) just before the two phenylalanine residues. In the other structure with a fully built arm this helix, while recognizable, is noticeably elongated, and remarkably, in our case this helix is completely deformed and unrecognizable as a helix (Fig. 4C).

**Figure 5:**
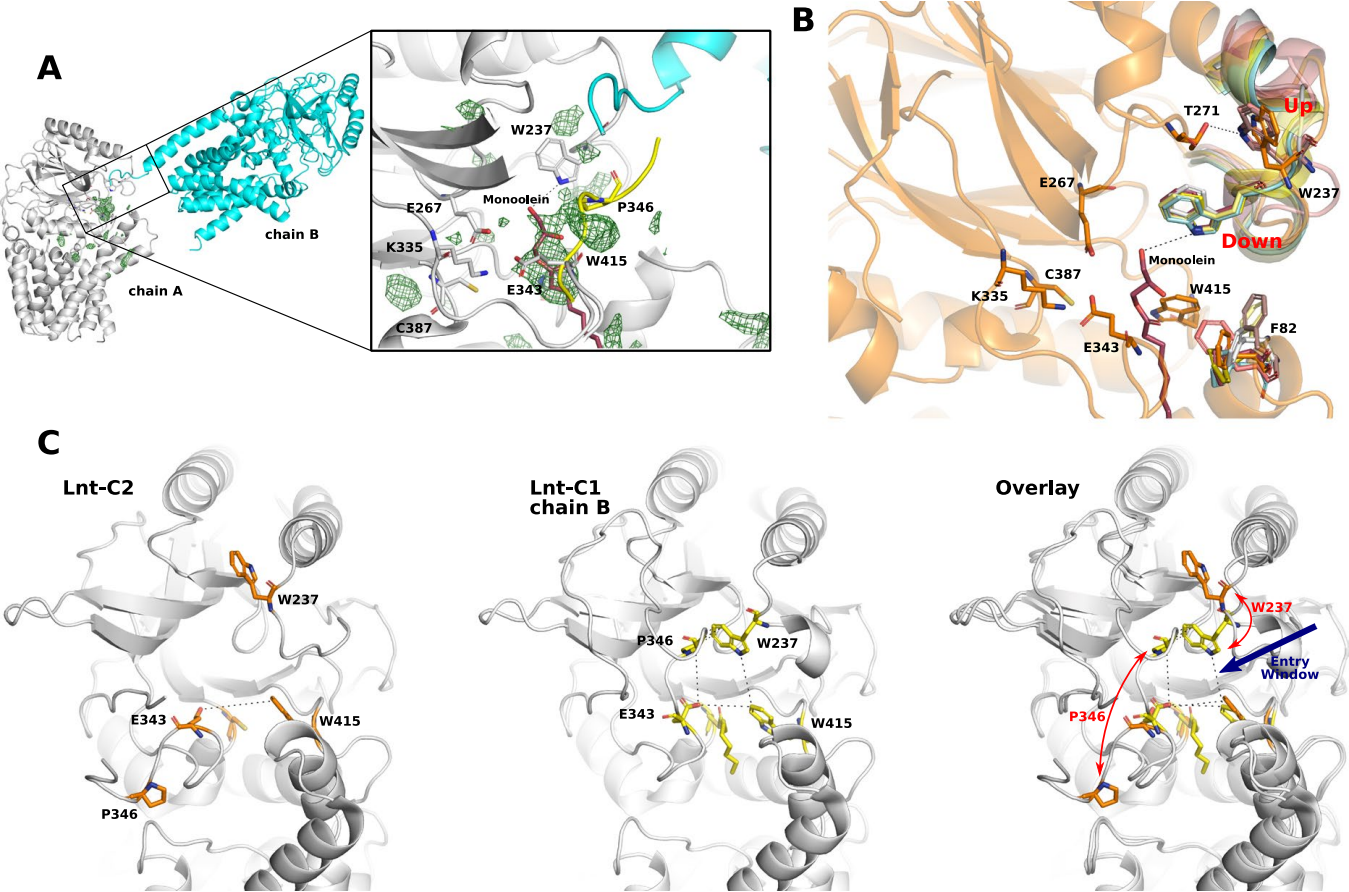
Conformational changes in the flexible loops. (**A**) The asymmetric unit of Lnt-C1. A portion of the C-terminus from chain B (cyan) is positioned at the entry to the substrate portal of chain A (white). Green mesh represents unknown positive electron density contoured at 2.5σ at the entry to the substrate portal. Zoom of boxed region: Overlay of monoolein (maroon, pdb 5VRG) and a piece of the flexible 345–365 loop (yellow, pdb 5XHQ) in the same position as unknown density. The essential W237 is in close coordination. (**B**) Position of W237 in the available crystal structures. In coordination with T271 when in the up position. In coordination with a lipid substrate when in the down position. (**C**) View looking into the large access cavity. Lnt-C2 has the W237 in an upward conformation and the long arm containing P346 in the membrane creating a very open cavity (left). A more restricted access cavity as seen in the thioester intermediate and 5XHQ with W237 in a downward position and the long arm containing P346 above the membrane (middle). Overlay of the two conformations illustrating the changes in the conformation of W237 and P346 (right).

In total, 3 conformations of this arm have now been observed (Fig. 4B) demonstrating its flexibility. In all cases where Lnt was crystallized in a more physiological environment using the LCP technique, the arm is positioned roughly parallel to the membrane interface. Molecular dynamics simulation^16^ have suggested that this arm interacts strongly with the lipid bilayer consistent with the observation that all of the structures solved using the LCP method had this arm in a position that would be embedded in the monoolein membrane. However, when crystallized using the vapor-diffusion method and thus in the absence of a stabilizing lipid bilayer, (pdb id 5XHQ and chain B of Lnt-C1) this arm is noticeable higher than the predicted membrane interface. In both cases, the movement of this arm above the membrane places a portion of this arm (in particular residue P346) into close proximity to the W237 on the opposite side of the large access cavity that could potentially help protect the active site when in the highly reactive thioester state. The only outlier to this trend is the protomer in chain A of Lnt-C1. Despite crystalizing using the vapor diffusion method its arm is positioned roughly parallel to the membrane similar to those crystalized using the LCP method. This might be explained by the arrangement of the protomers within the asymmetric unit (Fig. 5A). The detergent micelle of the chain B protomer within the asymmetric unit could be holding this arm down in this position.

### Movement of W237 mediates potential substrate binding inside the cavity

As mentioned above Lnt-C2 crystallized using the lipidic cubic phase method with 1 molecule in the asymmetric unit. Similar to chain A of Lnt-C1 the full 345–365 arm could not be built but appears to extend parallel to the membrane similar to chain A of Lnt-C1 and other Lnt structures crystallized using the LCP method. The crystal structure of Lnt-C2 is remarkable in the fact that despite crystallizing in the lipidic cubic phase, it’s substrate binding portal is devoid of any bound substrates. In this crystal form, despite high B-factors in this area, W237, located on the opposite side of the access cavity to E343, can be seen in the upward position coordinated to T271 (Fig. 5B, 5C-left panel). Also, despite crystallizing in the presence of 400 mM Ammonium phosphate, no phosphate molecules could confidently be built into the model. In particular, Noland *et al*.^16^ suggest a phosphate binding site at W237 based on binding of a chloride ion and a molecule of monoolein in that position, however we do not see density to support binding in this position. Moreover, the loop containing W237 has by far the highest B-factor of the entire protein, if phosphate or other molecules are indeed bound there, a stabilizing effect, resulting in lower B-factors would be expected. Although it cannot be ruled out that phosphate alone cannot bind here, but requires the presence of a coordinating lipid or other molecule.

As mentioned Lnt-C1 crystallized using the vapor diffusion technique with two molecules in the asymmetric unit. As shown in Fig. 5A, although likely due to crystal lattice contacts, the two molecules are arranged within the asymmetric unit in such a way that might give insights into one possible mode of apolipoprotein docking to Lnt. The C-terminus of chain B is in close proximity to the essential W237 at the entry of the substrate portal of chain A (zoomed box Fig. 5A). The position of the protomer and angle of the C-terminus of chain B to the substrate portal of chain A appears to be consistent with docking simulations carried out by Wiktor *et al*.^9^ of di- and triacylated FSL-1 onto Lnt. While at this point the protomer arrangement is merely an observation, the similarities between the docking simulations of Wiktor *et al*.^9^ are compelling. Also, at the entry to the substrate portal, unexplained density is observed. Overlay of the pdb 5VRG places the head group of a molecule of monoolein into this density that would be coordinated to the nitrogen atom (NE1) of W237. Similarly, overlay of the pdb 5XHQ places a piece of the flexible 345–365 loop near this density, in particular P346 that could also interact with W237 (Fig. 5).

W237 appears to be positioned at the entry of this large cavity leading to the active site triad. An overlay of all available crystal structures results in W237 in 2 alternate positions: an upward position interacting with T271 pointing away from the substrate portal, and a downward position pointing into the substrate portal allowing interaction with substrates (either protein or lipid) (Fig. 5B). In the other crystal structures where W237 is in the upward position (pdb 5N6H and 5N6L) no substrates are seen in the substrate portal. In all cases where W237 is in the downward position, it is coordinated with a potential substrate (a lipid and/or peptide in the case of 5VRG, 5VRH, 5XHQ, Lnt-C1). This suggests that substrate binding in the entry portal triggers movement to the downward position of W237, potentially triggering catalysis. If measuring from the NE1 atom of the tryptophan sidechain, this residue has a range of movement of ~8.5 Å. It has also been suggested that F82^16^, that is positioned opposite W237 also at the entrance to the portal acts as a potential gatekeeper. This cannot be ruled out, although unlike W237, an overlay of the available structures does not reveal a clear trend in the presence or absence of a substrate (Fig. 5B).

## Discussion

Lnt performs the last crucial step of lipoprotein maturation in Gram-Negative bacteria by N-acylating the terminal cysteine of incoming apolipoproteins to form the final mature lipoprotein. As described above this occurs via a two-step ping-pong mechanism where the first step is the acyl transfer of the phospholipid substrate to create a thioester acyl linkage on the active site cysteine. In the second step, with Lnt now in its thioester-acyl intermediate state, the alpha-amino group at the N-terminus of the incoming apolipoprotein attacks the thioester linkage to transfer the acyl chain to produce the final mature lipoprotein.

Mass spectral analysis of both Lnt-C1 and Lnt-C2 identified a palmitoyl modification at position C387. However, we only see evidence in the form of extra electron density extending off the C387 consistent with the thioester-acyl intermediate state in chain B of Lnt-C1. Despite seeing a significant percentage of covalently modified enzyme no electron density consistent with this linkage is seen in the structures presented by Noland *et al*.^16^, in the other solved structures of Lnt^9,17^, or in crystal form Lnt-C2 of this study. This is perhaps not surprising due to the highly labile nature of the thioester linkage and the fact that the active site sits at the back of a large cavity that is highly accessible to the environment. Due to this accessibility, the entry of any number of chemicals used during the purification and crystallization process could deacylate the thioester intermediate in the absence of the second substrate. This is especially relevant for Lnt-C2 that crystallized in an open conformation that exposes the C387 to the surrounding environment and took 6 weeks to crystallize.

In the same protomer that the thioester-acyl intermediate is found with the highest occupancy, the long 345–365 arm is seen at an acute ~60° upward angle to where the membrane is predicted to be. Although unable to build a complete arm Lu *et al*.^17^ also see this arm in an upward angle (albeit at a less acute angle) and refer to it as the lid loop. It is tempting to speculate if the positioning of this arm and the trapping of the thioester-acyl intermediate are connected. As aptly named by Lu *et al*.^17^ this arm could be acting as a protective lid to the otherwise highly open active site when in this upward position sheltering this reactive intermediate state from the surrounding environment and deacylation. In chain A of Lnt-C1 and Lnt-C2 this arm is positioned at the membrane interface creating a highly open active site cavity which allow the entry of any number of chemicals used during the purification and crystallization process deacylating the thioester explaining why this extra electron density is not seen (or at a very low occupancy) despite mass spectral evidence. Although the positioning of this arm in Lnt-C1 is likely due to crystal lattice contacts, it at the least demonstrates its flexibility and range of potential motion. In fact, the position of P346 is very close to the P346 of 5XHQ when overlaid could suggest that at least this portion of the arm could be in a biochemically relevant position, while the position of the remaining portion that is pinned at this extreme angle is likely the result of crystal lattice contacts.

No large movements of the active site residues are seen when comparing the palmitoylated vs non- palmitoylated active sites. This is consistent with the structure and proposed ping-pong mechanism of Lnt. Similarly, no large movements of the active site residues are seen in the recently reported structure of the human DHHC palmitoyltransferase when its active site cysteine is palmitoylated^22^ and binding of monoolein close to the Lnt active site^16^ did not cause any rearrangement. The bulk and stability created by extensive hydrogen bonding within the nitrilase fold creates a very rigid active site, and with the active site sitting at the back of the large open cavity that allows the easy entry and exit of substrates there is ample room to accommodate these substrates without the requirement for the inefficient rearrangement of the active site during enzyme turnover. However, although difficult to judge given the resolution limits there could be a small movement of the E343 side chain in the thioester intermediate state that could potentially help stabilize it. Consistent with that, E343 is an essential residue and is highly conserved in Lnt homologs and has been implicated through mutational studies^11,18,20^ in the first step of the Lnt reaction. Remarkably, the position of this residue overlaps very well in the various crystal structures despite a large difference in the position of the 345–365 arm^19^. The fact that the E343 is positioned near the active site and at the hinge of this arm could suggest some type of long-range communication between the active site and the outer tip of the 345–365 arm.

Also, the observation of a potential transient (dis)appearance of a short helix in this arm is fascinating. Although the unwinding of transmembrane helices has been reported in different functional states of Rhodopsin^23^ and it has recently been shown in Spectrins that helices can act to buffer force via a spring-like mechanism^24^ the loss of secondary structure elements seen here could simply be the result of crystal lattice contacts at the outer regions of the arm. At this time, other than pointing out this observation, we cannot say if this is physiologically relevant or not. F358 has been shown to be essential for the second step of the Lnt reaction^18^ and the propensity of this arm to form crystal contacts with this residue in our C1 construct and the only other fully built arm^9^ could suggest a role in potentially helping to direct and stabilize incoming substrate apolipoprotein molecules; but further experiments would need to be performed in order to prove or disprove this.

Similar to F358, mutational studies have shown that W237 is essential for only the second step of the Lnt mechanism^20^, i.e. the transfer of the acyl chain from C387 to the N-terminal cysteine of the lipoprotein. These studies^16,20^ have shown W237A variants could not rescue growth in cells lacking the *lnt* gene. It is located on a small highly mobile loop directly on the opposite side of the cavity to E343 that sits at the joint of the 345–365 arm. Based on overlay of the available structures we can see two distinct conformations of W237 separated by ~8.5 Å that seems to be correlated to the absence (up position) or presence (down position) of a potential substrate. W415 and E343 that appear to be relatively rigid are located at the narrowest part of the access cavity. The movement of W237 to the downward conformation positions it in proximity to these residues as well as P346 of the long 345–365 arm when this arm is above the membrane potentially creating a binding site for the peptide and/or lipid of the incoming apolipoprotein (Fig. 5C). When W237 is in the upward conformation and the 345–365 arm is positioned in the membrane this potential binding site is destroyed, thus destabilizing any Lnt-substrate complex and creating a highly open access cavity as seen in construct C2. In the thioester intermediate state of Lnt, W237 is also in the downward conformation; however the acyl chain of the thioester is ~8 Å away from the NE1 of W237, and thus too far way to have any (de)stabilizing or other effects. In both the thioester intermediate and the structure (5XHQ) of Lu *et al*.^17^ P346 is above the membrane and in close proximity to W237 when it is in the downward position. In these positions, P346 of the long 345–365 arm and W237 of the small loop could help shield the highly reactive intermediate from the environment while at the same time create a restricted, and potentially selective window (Fig. 5C) possibly allowing only the entry of the N-terminus of the apolipoprotein.

Taken together, we suggest that the short loop containing W237 might help to locally stabilize the N-terminal tail from the opposite side to allow transfer of the palmitoylate from the C387 to the apolipoprotein to produce the final mature lipoprotein. When W237 is in the downward conformation and the 345–365 arm is in a conformation above the membrane a window might be created with the relatively stable essential residues E343 and W415. The stable E343 and W415 could essentially create the bottom of a window pane that is always present at the narrowest part of the cavity, while W237 and P346 could associate to form the top portion of the window frame. In this conformation the cavity is restricted, allowing access to the reactive thioester state to potentially only N-terminals of apolipoproteins (Fig. 5C).

Mutations to residues that make up the 4 corners of our proposed window underscore the importance of this region of the protein. A large mutational study by Vidal-Ingigliardi *et al*.^20^ found that cells lacking the *lnt* gene could not be rescued with plasmid expressed W237A or E343A variants of Lnt and even suggested that these 2 residues open and close upon the binding and release of phospholipid and/or apolipoprotein. This is consistent with our findings, except while W237 opens and closes, E343 stays relatively rigid. Vidal-Ingigliardi *et al*.^20^ also found plasmid expressed P346A was nonfunctional at higher growth temperatures^20^. This could also be consistent with our findings; with P346 located on the highly mobile arm, the higher growth temperature could in fact input too much energy and create too much movement and thus prevent its association to form the top part of the window frame. Lastly, the W415A variant also failed to rescue growth in cells lacking the *lnt* gene and in fact expressed protein was undetectable^16^ suggesting this residue is critical in overall Lnt folding and stability. This is also consistent with our data and the other available structures. W415 and E343 form the bottom of the window and would bring some stability to an otherwise highly dynamic region of the protein.

As we focus here on the more dynamic aspects of Lnt, we cannot help but notice the striking contrast between the highly rigid inner core housing the active site triad and the highly dynamic outer arms leading to that active site. While one side of the nitrilase fold; referred to as the backend^9^; is rounded and otherwise featureless, the other side is splayed open like a hungry octopus opening its tentacles to expose its mouth (Fig. 2). Similarly, like a hungry octopus, Lnt could use these highly dynamic ‘tentacles’ to capture and close around and stabilize incoming apolipoproteins. Once the second step of the Lnt reaction occurs, the ‘tentacles’ of Lnt could then be loosened from the mature lipoprotein allowing its release. The data presented focuses mainly on the second step of the Lnt reaction where we provide new insights that could point to important substrate-induced protein dynamics for active site access and catalysis in Apolipoprotein N-acyltransferase. It is clear from our analysis that the reaction of Lnt is a highly complex mechanism requiring the movement of multiple highly dynamic loops and side chains working in concert to produce a final mature lipoprotein. Although there are still many unanswered questions about the exact molecular details, we hope together with our review of the other recently published structures and mutational studies, this new data provides a solid starting point for future experiments.

## Methods

### Cloning, expression and protein purification

The full length Lnt gene was amplified from *E*. *coli* K12 (DH5α) genomic DNA using standard PCR techniques with the primers 5′ ACTCAGCTCGAGATGGCTTTTGCCTCATTAATTGAACGC 3′ and 5′ ATCGACGAATTCTTTACGTCGCTGACGCAGACTCATCAAC 5′ and inserted into a modified pWaldo vector^25^ lacking the GFP reporter for a C-terminal His_8_-tag construct (C1)^26^ using the restriction sites *XhoI* and *EcoRI*. The second construct (C2) was constructed using the primers 5′ ACTCAGCTCCATATGGCTTTTGCCTCATTAATTG 3′ and 5′ ACTGACGAATTCTTATTTACGTCGCTGACGCAG 3′ and inserted into the pET-28 vector (Novagen) containing a N-terminal TEV protease cleavable His_6_-tag using the restriction sites *NdeI* and *EcoRI*.

For large-scale protein purifications, a plasmid containing Lnt was transformed into chemically competent *E*. *coli* C41. A pre-culture from a single colony was grown overnight at 37 °C in LB broth containing 50 µg/mL kanamycin, which was then used to inoculate 1.5 L LB broths in a LEX bioreactor (epiphyte3). Since in both constructs protein expression is driven from a T7 promoter, cells were grown at 37 °C until an OD_600_ = 1.0 prior to the induction of protein expression with 0.4 mM IPTG at ambient temperature for 4 hours. Cells were then harvested *via* centrifugation at 6000 g for 15 minutes and frozen at −20 °C. Frozen cells were thawed and suspended in lysis buffer (50 mM TRIS pH 8.0), and lysed by passage of at least 3 times through an emulsifex cell disrupter (Avestin). The lysate was centrifuged at 16000 g for 40 minutes to pellet cell debris. Membranes were then isolated by ultra-centrifugation of the cleared lysate at 40000 g for 40 minutes; washed one time with a washing buffer (500 mM NaCL, 50 mM TRIS pH 8.0); ultra-centrifuged again, and finally suspended in buffer containing 300 mM sucrose, 20 mM TRIS, pH 8.0, and frozen at −80 °C.

Purified membranes were diluted 20 times with buffer A (50 mM TRIS pH 8.0, 400 mM NaCl, 5% glycerol, 10 mM imidazole, 5 mM β-mercaptoethanol) and solubilized with 1% n-Dodecyl-β-D-Maltopyranoside (DDM) (Anatrace) at 4 °C for 90 minutes on a mixer, followed by centrifugation at 40000 g for 45 minutes to remove unsolubilized membranes. The resulting supernatant was incubated at 4 °C on a mixer with Ni-NTA resin (Qiagen) equilibrated with buffer A supplemented with 0.05% DDM for 90 minutes. The resin was washed with 100 mL buffer B (50 mM TRIS pH 8.0, 200 mM NaCl, 5% glycerol, 25 mM imidazole, 5 mM β-mercaptoethanol, 0.04% DDM), followed by a second 100 mL of buffer C (50 mM TRIS pH 8.0, 200 mM NaCl, 5% glycerol, 50 mM imidazole, 5 mM β-mercaptoethanol, 0.04% DDM). The C-terminal construct was eluted from the Ni-NTA resin with buffer D (50 mM TRIS pH 8.0, 150 mM NaCl, 5% glycerol, 250 mM imidazole, 5 mM β-mercaptoethanol, 0.4% n-Nonyl-β-D-Maltopyranoside (NM). The eluted protein was concentrated with a 100 kDa MWCO ultrafiltration spin column (Vivaspin) to less than 5 mL and further purified by injection into a HiLoad 16/60 Superdex 200 column (GE Healthcare) equilibrated with buffer E (25 mM TRIS pH 8.0, 100 mM NaCl, 5% glycerol, 0.5 mM TCEP, 0.35% NM). The purified protein eluted as a single peak at approximately 73 mL volume and the appropriate fractions were pooled and concentrated with a 100 kDa MWCO ultrafiltration spin column (Vivaspin) to 15 mg/mL. The final purified protein was snap frozen in liquid nitrogen, and stored at −80 °C if not used immediately for use in crystallization assays.

The N-terminal TEV-cleaved construct (C2) was purified as described above except the protein was eluted from the Ni-NTA resin with buffer F (50 mM TRIS pH 8.0, 150 mM NaCl, 5% glycerol, 250 mM imidazole, 5 mM β-mercaptoethanol, 0.03% DDM). The eluted protein was concentrated with a 100 kDa MWCO ultrafiltration spin column (Vivaspin) to less than 5 mL, diluted with buffer G (25 mM TRIS pH 8.0, 100 mM NaCl, 5% glycerol, 0.5 mM TCEP, 0.02% DDM) tenfold and incubated at 4 °C with TEV protease containing a His_6_-tag in a 1:10 ratio overnight on a mixer. The next morning, the mixture was incubated at 4 °C with Ni-NTA resin equilibrated with buffer G for 2 hours on a mixer. The Ni-NTA resin was collected with a gravity flow column and the flow-through was concentrated with a 100 kDa MWCO ultrafiltration spin column (Vivaspin) to less than 5 mL and further purified by injection into the HiLoad 16/60 Superdex 200 column (GE Healthcare) equilibrated with buffer G. The TEV-cleaved purified protein eluted as a single peak at approximately 70 mL volume. The appropriate fractions were pooled and the final purified protein was concentrated with a 100 kDa MWCO ultrafiltration spin column (Vivaspin) to 15 mg/mL, snap frozen in liquid nitrogen, and stored at −80 °C if not used immediately for use in crystallization assays.

### Crystallization

For crystallization trials with the C-terminal protein at 15 mg/mL was used to screen for initial crystal hits using the commercially available screens Memgold and Memgold2 (Molecular Dimensions). Crystallization trials were set up using a Mosquito liquid dispenser robot (ttplabtech) into 96 well, 2 drop, vapor diffusion sitting drop plates with 50 µL reservoir solution and 1:1 and 2:1 protein:precipitate ratios with a final drop volume of 300 nL. The final optimized condition was 25% PEG2000MME, 100 mM Na cacodylate pH 6.4, 40 mM MgCl_2_ grown at 18 °C, with a drop ratio of 1:1.75 protein:precipitate. Crystals grew to about 0.3 × 0.2 × 0.2 mm in about 2 weeks and were loop-harvested and snap-cooled in liquid nitrogen without added cryo-protectant.

For crystallization trials with the N-terminal, TEV cleaved protein, Lnt was reconstituted into the lipidic cubic phase using standard techniques^27^. The protein solution at 15 mg/mL was homogenized with monoolein (9.9 MAG) in a coupled syringe mixing device using two volumes of protein solution and three volumes of lipid. Initial crystallization trials were set up by transferring 50 nL of the protein-laden mesophase onto a silanized 96-well glass sandwich plate followed by 800 nL of precipitant solution using a LCP-Mosquito liquid dispenser robot (ttplabtech) and stored at 18 °C for crystal growth. Optimized crystals were obtained with 36% PEG200, 400 mM Ammonium phosphate dibasic, 100 mM HEPES pH 7.2. Best diffracting crystals were obtained by storing the glass plate at 10 °C for 3 weeks, followed by transfer to 4 °C for an additional 3 weeks. Crystals were loop harvested and snap frozen in liquid nitrogen without the addition of cryo-protectant after 6 weeks of growth.

### Data collection, processing, and structure determination

During the optimization process X-ray diffraction experiments were carried out at various synchrotron light sources across Europe, namely the ESRF, DIAMOND, BESSY II, and the SLS. The final high resolution datasets were collected at the SLS, beamline X06SA-PX. Data was processed with XDS^28^ and AIMLESS^29^. The structures were solved by molecular replacement with the pdb model 5N6L as a search model searching for 2 molecules in the asymmetric unit for the C-terminal construct and 1 molecule for the N-terminal TEV cleaved construct using the PHENIX suite of programs^30^ with final model building in Coot^31^. The palmitoylated C387 was modeled using the crystal structure of a palmitoylated TEAD3 transcription factor^21^ as a base. Data collection parameters and refinement statistics are reported in Table 1. Figures were generated using PyMOL^32^ or UCSF Chimera^33^.

**Table 1:** Data Collection and Refinement Statistics.

	Lnt-C1 PDB: 6NWR	Lnt-C2 PDB: 6Q3A
**Data Collection**		
X-ray source	SLS, X06SA-PX	SLS, X06SA-PX
Wavelength (Å)	1.00	1.00
Space group	P2_1_2_1_2_1_	P6_4_22
Cell Dimensions		
a, b, c (Å)	72.164, 136.989, 220.890	80.725, 80.725, 442.575
α, β, γ (°)	90.0, 90.0, 90.0	90.0, 90.0, 120
Resolution (Å)	48.47-3.5 (3.6–3.5)	46.89-3.01 (3.2–3.01)
Completeness	99.8 (99.7)	99.7 (100)
Redundancy	7.3 (7.3)	20.9 (19.2)
*I*/σ(*I*)	7.49 (1.05)	13.97 (0.59)
R_meas_	0.180 (2.10)	0.194 (4.93)
CC_1/2_	0.993 (0.497)	0.999 (0.232)
**Refinement**		
Resolution (Å)	48.56-3.5	46.89-3.1
No. of unique reflections	28382 (2802)	29269 (2907)
R_work_/R_free_	0.26/0.28	0.26/0.30
No. of atoms	7988	3920
Protein	7885	3877
Ligands	103	43
B-factors (Å^2^)		
Protein	109	120
Ligands	163	129
RMSD		
Bond length (Å)	0.004	0.004
Bond angles (°)	0.66	0.96
Ramachandran plot (%)		
Favored	93.94	94.26
Allowed	6.06	5.74
Outliers	0.00	0.00

Values in parentheses are for the highest-resolution shell. RMSD, root-mean-square deviation.

### Mass spectrometry

20 µg of purified Lnt-C1 and Lnt-C2 were digested with trypsin without reduction or alkylation and then purified using a Pierce C18 Spin Columns (Thermo Scientific) and dried. The dried peptides were resolved in 60 μL of 0.1% formic acid and further diluted 4 times just prior to nano-liquid chromatography - tandem mass spectrometry (LC-MS/MS). The resulting peptides were separated in reversed-phase on a C18-column and electrosprayed online to a QExactive Plus Orbitrap mass spectrometer (Thermo Finnigan) with a 35 min gradient. Tandem mass spectrometry was performed by applying higher energy collisional dissociation (HCD) spectra of the same precursor. Database searches were performed using the Sequest algorithm, embedded in Proteome Discoverer 1.4 (Thermo Fisher Scientific) with search parameters set to the enzyme trypsin (Variable modifications were Oxidation, Deamidated and Palmitoyl). The search criteria for protein identification were set to at least two matching peptides of 95% confidence level per protein.

## Data Availability

The atomic coordinates and structure factors have been deposited in the Protein Data Bank under accession codes 6NWR and 6Q3A.
